# Emerging Vector-Borne Diseases – Incidence through Vectors

**DOI:** 10.3389/fpubh.2014.00267

**Published:** 2014-12-02

**Authors:** Sara Savić, Branka Vidić, Zivoslav Grgić, Aleksandar Potkonjak, Ljubica Spasojevic

**Affiliations:** ^1^Scientific Veterinary Institute, Novi Sad, Serbia; ^2^Department of Veterinary Medicine, Faculty of Agriculture, University of Novi Sad, Novi Sad, Serbia

**Keywords:** emerging diseases, zoonoses, vector-borne diseases, One Health, ELISA

## Abstract

Vector-borne diseases use to be a major public health concern only in tropical and subtropical areas, but today they are an emerging threat for the continental and developed countries also. Nowadays, in intercontinental countries, there is a struggle with emerging diseases, which have found their way to appear through vectors. Vector-borne zoonotic diseases occur when vectors, animal hosts, climate conditions, pathogens, and susceptible human population exist at the same time, at the same place. Global climate change is predicted to lead to an increase in vector-borne infectious diseases and disease outbreaks. It could affect the range and population of pathogens, host and vectors, transmission season, etc. Reliable surveillance for diseases that are most likely to emerge is required. Canine vector-borne diseases represent a complex group of diseases including anaplasmosis, babesiosis, bartonellosis, borreliosis, dirofilariosis, ehrlichiosis, and leishmaniosis. Some of these diseases cause serious clinical symptoms in dogs and some of them have a zoonotic potential with an effect to public health. It is expected from veterinarians in coordination with medical doctors to play a fundamental role at primarily prevention and then treatment of vector-borne diseases in dogs. The One Health concept has to be integrated into the struggle against emerging diseases. During a 4-year period, from 2009 to 2013, a total number of 551 dog samples were analyzed for vector-borne diseases (borreliosis, babesiosis, ehrlichiosis, anaplasmosis, dirofilariosis, and leishmaniasis) in routine laboratory work. The analysis was done by serological tests – ELISA for borreliosis, dirofilariosis, and leishmaniasis, modified Knott test for dirofilariosis, and blood smear for babesiosis, ehrlichiosis, and anaplasmosis. This number of samples represented 75% of total number of samples that were sent for analysis for different diseases in dogs. Annually, on average more then half of the samples brought to the laboratory to analysis for different infectious diseases are analyzed for vector-borne diseases. In the region of Vojvodina (northern part of Serbia), the following vector-borne infectious diseases have been found in dogs so far borreliosis, babesiosis, dirofilariosis, leishmaniasis, and anaplasmosis.

## Introduction

Vector-borne diseases use to be a major public health concern only in tropical and subtropical areas, but today they are an emerging threat in continental countries also. Nowadays, in intercontinental countries, there is a struggle with some emerging diseases, which have found their way to appear through vectors. Vector-borne zoonotic diseases occur when vectors, animal hosts, climate conditions, pathogens, and susceptible human population exist at the same time, at the same place. Global climate change is predicted to lead to an increase of vector-borne infectious diseases and disease outbreaks. It could affect the range and population of pathogens, host and vectors, transmission season, etc. Reliable surveillance for diseases that are most likely to emerge is required. There are countries where environmental conditions are not so favorable for certain vector populations, but immigration allows them to persist (1). Vector-borne pathogens have a considerable impact on human and animal health. Newly emerging pathogens and increasing case numbers of endemic diseases have received considerable public attention in Europe recently. Vector-borne diseases are a matter of Public Health, and therefore, it is important to establish a systematic approach for the understanding, analysis, assessment, communication, and the management risk associated with vector-borne diseases. Risk assessment of vector-borne diseases, however, is influenced by the fact that the infection depends on many factors, the probability of infection in human beings and animals, and the spread of pathogen, or vectors. These factors are climate change, change in human beings living habits, agricultural land usage, individual human behavior, travel, and global trade (2).

The complex epidemiology of vector-borne diseases creates significant challenges in the design and delivery of prevention and control strategies, especially in sight of rapid social and environmental changes. Many diseases are specially constrained, for example, vector-borne and zoonotic diseases occur where and when vectors, animal hosts, pathogens, and susceptible human populations overlap (3). Global climate change is predicted to lead to an increase of infectious disease outbreaks. Reliable surveillance for diseases that are most likely to emerge is required. Climate changes could affect the range and population size of pathogens, hosts and vectors, the length of the transmission season, and the timing and persistence of out breaks (4). If there are outbreaks of infectious diseases that are considered to be eradicated from before or that are totally under control, they are called emerging infectious diseases (5). Emerging infectious diseases can be defined as infections that have newly appeared in a population, or are rapidly increasing in incidence or geographic range. Many of these diseases are zoonoses (6). From all the causative agents of emergent infectious diseases, 60–70% of them have a zoonotic potential (7). Zoonoses are infectious diseases, which can be transferred from animals (mammals) to human beings. Maps of expected distributions of vector existence are often presented as a risk of exposure to a pathogen. Global climate change is predicted to lead to an increase of vector-borne infectious diseases and disease outbreaks. It could affect the range and population of pathogens, hosts and vectors, transmission season, etc. Reliable surveillance for diseases that are most likely to emerge is required.

The factors of emergence are the following: changes in ecology, changes in demography and human behavior, changes and adaptations of microorganisms, improvement in technology, and changes in industry, international transport and trade, and incompliance of public health measures. Changes in ecosystem may lead to the increase of population in natural hosts, or vectors for certain emerging infectious disease. These factors are becoming increasingly prevalent, suggesting that infections will continue to emerge and probably increase. Strategy for dealing with this problem includes focusing of attention on promoting the emergence of diseases, especially in situations when animals and human beings are in contact and implementation of effective disease surveillance and control (6).

For practicing veterinarians, vector-borne diseases represent a constant challenge. The health of companion animals never played a more important role in a family life. It is expected from veterinarians to play a fundamental role in first of all prevention and then treatment of vector-borne diseases in dogs. Canine vector-borne diseases represent a complex group of diseases including anaplasmosis, babesiosis, bartonellosis, borreliosis, dirofilariosis, ehrlichiosis, leishmaniosis, and rickettsiosis. Some of these diseases cause serious clinical symptoms in dogs and some of them have a zoonotic potential with an effect to public health. Veterinarians are comfortable accepting that a multi-modal approach in disease diagnostics, including antigen and antibody testing combined with other diagnostic methods is ideal to diagnose some diseases. But, it is very important to highlight that increased detection of infection with, or exposure to, canine vector-borne diseases does not always confirm the disease cause. It is still the veterinarian who is responsible for the interpretation of the laboratory findings, having in mind clinical status, and response to the treatment. To optimize the clinical division, veterinarians in practice should always consider using panels that include serological and PCR assays in parallel to maximize their chances of detecting infection or exposure to canine vector-borne pathogens (8). Also, annual control of artropodes using ectoparasiticides with repellents, to block the interaction between vector and host is upon veterinarians to be recommended to the owners (9).

Vector-borne diseases such as tick-borne *Borrelia burgdorferi s.l*, tick- and flea-borne emerging and re-emerging *Rickettsia* species, and sandfly borne *L. infantum* are important infectious diseases of human and veterinary medicine across Europe (9). A tick species *Ixodes ricinus* is usually predominant among ticks originating form Serbia and is one of the most widely distributed. A significant presence of *B. burgdorferi sensu lato* was detected in *I. ricinus* ticks from Serbia, using dark field microscopy (10, 11). Also, multiple and mixed infections with different pathogens were found in different species of ticks in Serbia (12) with a highlight on *I. ricinus*, in which also mixed infections were found (13).

## Materials and Methods

During a 4-year period, from 2009 to 2013, a total number of 734 dog samples were analyzed for different diseases. For vector-borne diseases (borreliosis, babesiosis, ehrlichiosis, anaplasmosis, dirofilariosis, and leishmaniasis), 551 dog blood samples were analyzed in routine laboratory work, which makes 75% of total samples analyzed for different diseases. The analysis was done by serological tests – ELISA for borreliosis (Microgen IgG and IgM and Euroclone commercial ELISA set kits for detection of specific antibodies against *Borrelia*), leishmaniosis (Ingenaza commercial ELISA set kit for detection of specific antibodies against *Leishmania*), and dirofilariosis (IDEXX commercial ELISA set kit for detection of specific antibodies against *Dirofilria*). The procedure of analysis was done by the original manufacturer’s instructions. Diagnostic of dirofilariosis was also done with modified Knott test for dirofilariosis. Diagnosis of babesiosis, ehrlichiosis, and anaplasmosis was done in a stained blood smear. The samples were collected when dogs came for a routine check-up, or when they were sent for a certain disease detection because of the clinical symptoms that could be found in the animal. Some of the dogs had clinical symptoms, which could be identified as clinical symptoms for borreliosis, leishmaniosis, or dirofilariosis. Some of the samples were sent for a complete check-up, with a panel of all six diseases, or different combination of more then one, depending on the needs of the patient. Fast and snap tests for diagnostics of one or combination of pathogens were not used at any time.

## Results and Discussion

The number of samples analyzed for vector-borne zoonoses: borreliosis, babesiosis, ehrlichiosis, anaplasmosis, leishmaniosis, and dirofilariosis represented 75% of total number of samples that were sent for analysis for different diseases. Annually, on average, more then half of the samples brought to the laboratory for analysis on different diseases are analyzed for vector-borne diseases. In the region of Vojvodina (northern part of Serbia), the following vector-borne infectious diseases have been found so far in dogs: borreliosis, babesiosis, dirofilariosis, leishmaniasis, and anaplasmosis. For babesiosis, ehrlichiosis, and anaplasmosis a routine diagnostic procedure started only in 2012 in our laboratory, so the number of these samples was taken into consideration since that period.

In the same region, the following diseases have been diagnosed in human beings, so far borreliosis, dirofilariosis, and leishmaniosis (imported cases). Borreliosis exists as a common disease in human beings in Serbia. Several cases of dirofilariosis in human beings have been found during the last 4 years in Vojvodina and also cases of leishmaniasis have been found during the same period, but only as imported cases (unpublished data). Dirofilariosis has also been found in human beings Serbia during the last few years (14).

A total number of 551 dog samples were analyzed for different vector-borne diseases (borreliosis, babesiosis, ehrlichiosis, anaplasmosis, dirofilariosis, and leishmaniasis) in routine laboratory work. Total number of analyses done in these samples was 692. The results are presented in Tables 1 and 2.

**Table 1:** **Total number and positive findings in dog samples for vector-borne diseases during the period 2009–2013**.

	2009 (Positive %)	2010 (Positive %)	2011 (Positive %)	2012 (Positive %)	2013 (Positive %)
Borreliosis	13 (30.7%)	45 (24.4%)	30 (23.3%)	25 (16%)	77 (29.8%)
Babesiosis	–	–	–	17 (11.7%)	24 (12.5%)
Ehrlichiosis	–	–	–	30 (0%)	59 (0%)
Anaplasmosis	–	–	–	30 (0%)	59 (10.1%)
Leishmaniasis	5 (20%)	14 (14.28%)	53 (15.1%)	15 (13.3%)	133 (15%)
Dirofilariosis	7 (28.5%)	11 (27.7%)	15 (33.3%)	18 (38.8%)	101(24.7%)

**Table 2:** **Total number of analyses and positive findings for the study period 2009–2013**.

Total no. of analyses for the study period 2009–2013	
Borreliosis	190 (25.8%)
Babesiosis	41 (12.2%)
Ehrlichiosis	89 (0%)
Anaplasmosis	89 (6.74%)
Leishmaniasis	220 (15%)
Dirofilariosis	152 (27.6%)

During the observed period (2009–2013), 190 dogs were examined for Lyme borreliosis in routine work and 49 of them were found positive (25.8%). Seroprevalence for Lyme borreliosis in dogs was studied previously, for the earlier 3 year period (2006–2008) in a larger number of samples, with a randomly chosen group of samples and in the same region. Seroprevalence was found to be 25.81%. Also, the prevalence of Lyme borreliosis in ticks in northern part of Serbia was found to be 22.12% (15). In a study from Milutinovic et al., the highest prevalence rate found in different part of Serbia among ticks for *B. burgdorferi sensu lato* was 42.5%. So far, in Serbia presence of five *B. burgdorferi sensu lato* genospecies was found: *B. burgdorferi sensu stricto*, *B. afzelii*, *B. garinii*, *B. usitaniae*, and *B. valaisiana*. Also, co-infections were found in ticks with *B. burgdorferi sensu lato* and *A. phagocitophilum* (10).

Only 41 samples of dogs were examined for babesiosis. The reason for this small number of samples is that veterinarians perform the analysis for babesiosis themselves in their practices. Only complicated cases where they could not find positive result were sent for a conformation to our laboratory. Therefore, only five of those suspicious dogs with anemia were really positive for babesiosis.

Ehrlichiosis is an emerging disease coming to Serbia, with just a few positive findings within the country (16). In our study with the method of stained blood smear no positive samples were found.

Anaplasmosis is also a vector-borne disease new to veterinarians and owners in the region. Some fast tests are done in several practices, with very low incidence of the disease itself. In a study from different region of Serbia showed a prevalence of 5.40–6.60% (16). In our study, a similar prevalence was found and it was 6.74%.

The number of examined dog blood samples for leishmaniosis, during the period of study, was 220. The number of positive samples detected for leishmaniosis was 33, which makes 15%. A certain number of dogs have traveled abroad (Italy, Greece, or Montenegro) during a previous study period. Those dogs were examined after coming back to Serbia (21 dogs) for the presence of specific antibodies against *Leishmania*. From the total of 21 samples in 28.57%, a positive finding for leishmaniosis was detected and the dogs had clinical symptoms of leishmaniosis (17).

During the same period from 2009 to 2013, the number of 152 dogs was examined for dirofilariosis – with or without clinical symptoms, and in 69 samples (27.6%) the presence of microfilaria was detected. In a previous study period, the analysis was done on working and military dogs from a selected group, in the same region, where as a result a seroprevalence for dirofilariosis was found to be 18% (18).

Recently published data from Europe indicate that there is an urgent need for further research and more field studies about *Ixodes* ticks and tick-borne diseases. The epidemiology of *Ixodes* ticks, the dynamics of tick spreading into new habitats due to the change in climate and land use is not fully understood. Interaction in various habitats in Europe, between the vector, pathogen, and mammalian host (human beings and dogs) needs further research (9).

## Conclusion

Human and animal health is connected today into a One Health concept, which focuses on zoonotic pathogens emerging from wild life, domestic animals, and companion animals. A role of companion animals influence to public health is more important over the years, because the interaction and connection between human beings and pets are growing and getting stronger over time. There should be an interaction between veterinary and human medicine for the benefit of domestic, wild animal, and human health. It should always be in our minds that there is an interaction between human health and domestic animal and wild life health with global zoonotic disease pandemics and emerging infectious diseases, which came from these animal species. From the total of 734 dogs examined for different infectious diseases, 551 of them were tested for canine vector-borne diseases that exist in the region, in vectors and in human beings, and 692 analyses were done. From the total number of analysis, 23.4% of examined dogs blood samples were positive to one of the canine vector-borne diseases.

Borreliosis, babesiosis, leishmaniasis, dirofilariosis, and anaplasmosis are considered as major vector-borne infectious diseases that are shared by man and dogs in the region of the study, from the One Health concept point of view. There should be an interaction between veterinary and human medicine, with clinicians, researchers, and government working together for the benefit of domestic, wild animal, and human health and the global environment (19). Managing of canine vector-borne diseases requires a One Health approach and proper control of arthropod transmitted pathogens to both human beings and animals. This approach is only achievable when clinicians and researchers from different disciplines work joined. Legislative bodies nationally and on the European level should support this initiative (9).

## Conflict of Interest Statement

The authors declare that the research was conducted in the absence of any commercial or financial relationships that could be construed as a potential conflict of interest.
